# Design and integration of a problem-based biofabrication course into an undergraduate biomedical engineering curriculum

**DOI:** 10.1186/s13036-016-0032-5

**Published:** 2016-09-21

**Authors:** Ritu Raman, Marlon Mitchell, Pablo Perez-Pinera, Rashid Bashir, Lizanne DeStefano

**Affiliations:** 1 Department of Mechanical Science and Engineering, University of Illinois at Urbana-Champaign, Urbana, USA; 2 I-STEM Education Initiative, University of Illinois at Urbana-Champaign, Urbana, USA; 3 Department of Bioengineering, University of Illinois at Urbana-Champaign, Urbana, USA; 4 Center for Education Integrating Science Math and Computing, Georgia Institute of Technology, Atlanta, USA

## Abstract

**Background:**

The rapidly evolving discipline of biological and biomedical engineering requires adaptive instructional approaches that teach students to target and solve multi-pronged and ill-structured problems at the cutting edge of scientific research. Here we present a modular approach to designing a lab-based course in the emerging field of biofabrication and biological design, leading to a final capstone design project that requires students to formulate and test a hypothesis using the scientific method.

**Results:**

Students were assessed on a range of metrics designed to evaluate the format of the course, the efficacy of the format for teaching new topics and concepts, and the depth of the contribution this course made to students training for biological engineering careers. The evaluation showed that the problem-based format of the course was well suited to teaching students how to use the scientific method to investigate and uncover the fundamental biological design rules that govern the field of biofabrication.

**Conclusions:**

We show that this approach is an efficient and effective method of translating emergent scientific principles from the lab bench to the classroom and training the next generation of biological and biomedical engineers for careers as researchers and industry practicians.

**Electronic supplementary material:**

The online version of this article (doi:10.1186/s13036-016-0032-5) contains supplementary material, which is available to authorized users.

## Background

Training engineers to solve ill-structured real-world problems, including the unanticipated complications and conflicting constraints such problems entail, has long been a challenge to engineering educators. The traditional lecture-based approach of teaching a set curriculum, which focuses on solving well-structured problems, does not yield a skill set that is readily transferrable to engineering practice [1]. This finding has inspired a widespread transition to project-based learning in core engineering classes [2]. This new pedagogical model for teaching design requires students to identify and prioritize functional outcomes and employ different approaches to devising a solution, a closer mimic to the real-world engineering environment. While the importance of having design-integrated classes during every year of undergraduate education has been demonstrated in established engineering fields [3], the emerging field of bioengineering and biomedical engineering is still in the process of instituting discipline-wide standards and practices for undergraduate education [4].

Lab- and project-based approaches have successful records of teaching core engineering principles in interdisciplinary fields [5, 6]. This is especially important in biomedical engineering, which develops students’ fluency in a diverse breadth of topics, such as biomaterials and biomechanics, while equipping them with fundamental skills and topical depth in new biological frontiers, such as tissue engineering and synthetic biology [7–9]. In a discipline that is evolving as quickly as biomedical engineering, it is likely that an adaptive and inquiry-based approach that teaches the scientific method, in the context of cutting-edge biomedical research, will prove well suited to undergraduate education [10–12]. This manuscript outlines a modular and inquiry-based approach to teaching the fundamental principles of the burgeoning field of biofabrication, or “building with biology”, at the undergraduate level. Furthermore, we provide details regarding the semester-long course schedule and lab funding structure to enable other educators to adapt this course design for their needs. We hypothesize that this curriculum structure will effectively teach upper-level undergraduate students to design and execute experiments, preparing them for interdisciplinary careers as biomedical researchers and industry practicians.

## Methods

### Course structure

This study employed four teams of two students each, all senior undergraduates in the bioengineering program at the University of Illinois at Urbana-Champaign (UIUC) and enrolled in “BioE 306: Biofabrication Lab”. Since proficiency in aseptic mammalian cell culture technique is a pre-requisite to the labs in this course, enrollment was limited to students who had previously completed a cell culture lab course required of sophomores in the bioengineering program. Enrolled students were also required to complete online safety training as required by the Division of Research Safety (IBC-3923) and undergo a practical laboratory test on aseptic cell culture technique during the first week of class.

The course (BioE 306: Biofabrication) was structured as a series of four labs, each addressing a distinct aspect of biofabrication and tissue engineering, leading up to the final capstone project that required students to draw on skills learned in the prior labs (Tables 1 and 2; Additional file 1: Table S1). Students attended weekly lectures (50 min) and labs twice a week (2 h per session), and were expected to maintain their experiments during the week outside the regularly scheduled lab sessions. This course structure thus required a fully functional cell culture lab well-equipped with the supplies and reagents necessary for biofabrication experiments. A list of required lab supplies, as well as estimated total cost, is provided in Additional file 2: Table S2.

**Table 1:** Course schedule

Lab 1 (3 weeks)	Biocompatibility	Test the effect of different types of commonly used chemical compounds in 3D printers on cell viability
Lab 2 (3 weeks)	Lentiviral Transduction	Learn standard methods for delivering genes to cell lines and assessing transduction efficiency
Lab 3 (3 weeks)	3D Cell Culture in Hydrogel Scaffold	Culture muscle cells in 3D hydrogel scaffolds and quantify cellular adhesion, morphology, proliferation, and differentiation in 3D
Lab 4 (3 weeks)	Build a Walker Bio-Bot	3D print walking bio-bot powered by tissue engineered skeletal muscle and compute muscle force generation and bio-bot speed
Final Project (3 weeks)	Design and Build Your Own Bio-Bot	Design and build muscle-powered bio-bot to achieve a functional behavior relevant to an application in biomedical engineering

**Table 2:** Course objectives

Learning objective	Lab
Quantify cell viability of any type of tissue in culture	1, 2, 3, 4, 5
Deliver heterologous genes to cells in culture by infection using lentiviruses while understanding the safety concerns associated with viral transduction.	2, 3, 4
Grow mammalian cells in 3D environments and evaluation of phenotypic changes that occur as a result of modifications in the composition of the extracellular matrix	3,4
Design, build, and test skeletal muscle-powered biological machines	4,5
Evaluate professional and ethical concerns associated with the construction of synthetic biological machines	Ethics Discussion

The lectures for the first two labs were led by the course instructor, Prof. Pablo Perez-Pinera. The lectures for the third lab were led by the teaching assistant, Colin Castleberry. The lectures for the fourth lab and the final project were led by guest lecturer, Ritu Raman. Students also participated in an hour-long ethics discussion focused on building biological machines, led by Ritu Raman.

### Capstone design project

**Fig. 1:**
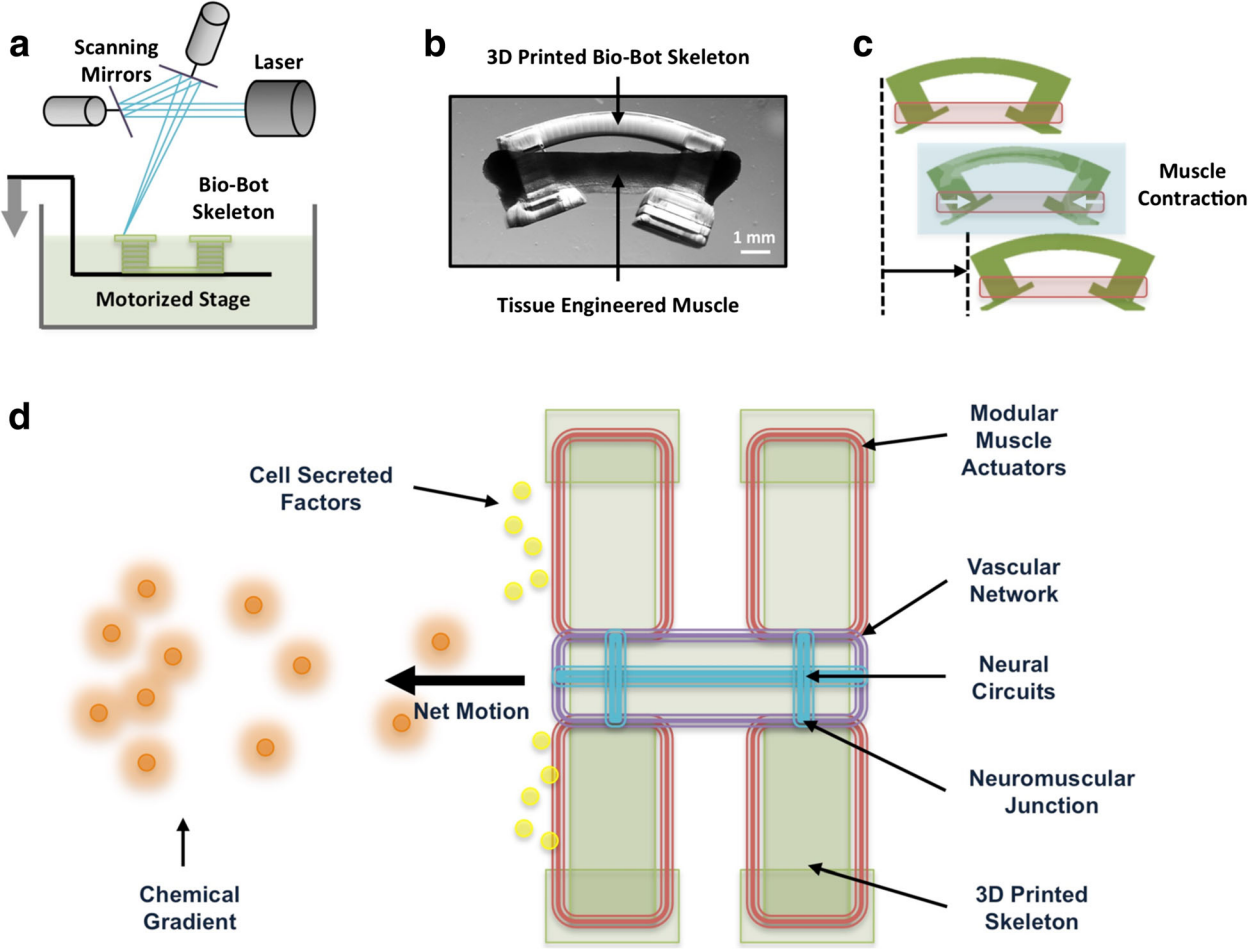
3D Printing Biological Machines. **a** Schematic of 3D printing apparatus used to fabricate bio-bot skeletons using a biocompatible polymer. **b** Image of 3D printed bio-bot coupled to tissue engineered skeletal muscle. **c** Electrical and optical signals are used to drive contraction of the tissue engineered muscle, with each contraction corresponding to a “step” forward. External signals can thus be used to control bio-bots to walk on 2D substrates. The direction of walking can be dictated by either the geometry of the skeleton or the region of muscle stimulated. **d** Future work on bio-bots could involve incorporating multiple tissue types (such as muscle, vasculature, neurons) to create robots that can sense, process, and respond to dynamic environmental signals in real-time. Shown in this schematic is a bio-bot that senses a harmful chemical gradient, walks toward it, and secretes biological factors to neutralize the toxin. This is just one of many potential applications for bio-bots in future

**Fig. 2:**
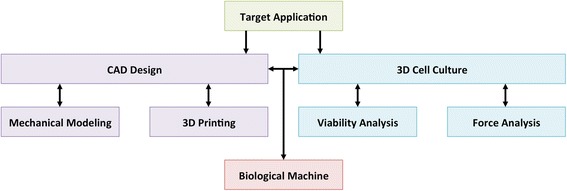
Biological Design Process for Capstone Project. Using the skills of 3D printing and 3D cell culture taught in the first four labs, students iteratively designed and built biological machines for specific target applications in the final project

The capstone final project required students to design, 3D print, and build a biological machine for any application of their choice. Such machines, which are composed of biological materials as well as synthetic materials, can utilize the dynamic sensing, processing, and actuation capabilities of biological tissues to perform controlled robotic functions (Figs. 1 and 2). Unlike traditional robots made of synthetic materials, such bio-integrated robots (bio-bots) could demonstrate the real-time adaptive behavior typical of biological systems and potentially be tuned to suit a variety of applications in health, security, and the environment [13–15]. The idea of forward engineering living tissues and using these tissues to power novel machines and systems has gained much attention in recent years, enabled by emerging manufacturing technologies such as 3D printing [16, 17]. This is the primary focus of the National Science Foundation Science and Technology Center EBICS (Emergent Behavior of Integrated Cellular Systems, NSF Grant CBET-0939511) [18]. Transitioning research in this pioneering field from the lab bench to the real world requires equipping the next generation of engineers and scientists with specific skills in biofabrication. Teaching the fundamental design principles and practices of “building with biology” is thus an important objective in training biomedical engineers for future careers in industry and academia. With this goal in mind, student teams were given 3 weeks to build muscle-powered bio-bots to target a specific challenge in biomedical engineering. Teams consulted with the course instructors during individual meetings in the first week to test and iterate the proposed bio-bot design for feasibility and functionality. After these initial design meetings, the instructors and teaching assistant assumed facilitator roles while students led their projects independently.

### Student assessment

The parameters considered for grading student performance were effort in the lab and participation (30 %), computer-aided design (CAD) project (10 %), team laboratory reports (four reports: 1.5, 4.5, 6, and 18 %) and a final presentation on the capstone design project (30 %) to course instructors, fellow students, and professors and graduate student researchers in related fields.

### Course evaluation

Students were assessed on the metrics listed in Table 3 in mid-course and end-of-course surveys. Additional metrics analyzed in the end-of-course survey are listed in Table 4. Students were also assessed on their responses to the open-ended questions listed in Table 5 (mid-course survey) and Table 6 (end-course survey). All raw data is presented in Additional file 3: Table S3, Additional file 4: Table S4, Additional file 5: Table S5 and Additional file 6: Table S6. Significance tests were conducted using Mann-Whitney *U* tests with *p* values signified in figure captions. This study was approved by the Institutional Review Board (Reference #12224). All students in the course signed consent forms before participating in this study.

**Table 3:** Metrics assessed in mid- and end-course surveys

Metrics	1	2	3	4	5
	Strongly Disagree	Disagree	Neutral	Agree	Strongly Agree
I expect/expected the course to introduce me to new topics and concepts					
The format of the course seems to be/was appropriate					
The lab reports are/were helpful for understanding the course material

**Table 4:** Metrics assessed in end-course survey

Metrics	1	2	3	4	5
	Strongly Disagree	Disagree	Neutral	Agree	Strongly Agree
Labs leading up to the final project helped me understand the final project better					
I felt well prepared for the final project presentation					
I feel that this course contributed greatly to my training in tissue engineering					
I believe the lab based format helped me learn tissue engineering (TE) concepts better than lecture based format					
I found value in working in teams on the labs and final project					
I would recommend this course to other students					
I enjoyed my experience in this course					
The course fulfilled my expectations

**Table 5:** Open-ended questions in mid-course survey

Metrics	Please explain/expand here
What aspects of the course are you most looking forward to?	
Do you want the biology to be explained more explicitly in the context of the material being taught?	
What are your thoughts about the level at which this course is being taught? (too advanced, just right, other)

**Table 6:** Open-ended questions in end-course survey

Metrics	Please explain/expand here.
Which aspect of the course helped you learn most efficiently?	
What aspects of the course did you find most useful?	
Was the biology explained explicitly enough to assist you with the other material being taught?	
Provide your thoughts about the level at which this course was taught (too advanced, just right, other)	

## Results and discussion

**Fig. 3:**
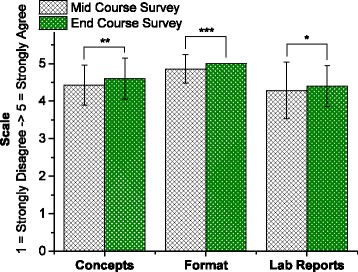
Comparison of Mid- and End-Course Survey Results. Student responses to questions listed in Table 3. Data is represented as mean ± standard deviation. Statistical significance is evaluated via a Mann-Whitney *U* test with *p* = 0.5 (*), 0.35 (**), 0.25 (***)

The quantitative survey metrics assessed over the course of the class all increased from the mid- to end-course time points, indicating an overall positive trend in the students’ course experience (Fig. 3, Additional file 3: Table S3).

When assessed during the mid-course time point, 100 % of students in the class confirmed that the course was being taught at an appropriate level. Additionally, 100 % of students in the class noted that they did not need the biology explained more explicitly in the context of the material being taught. These percentages did not change when assessed again at the end of the course, indicating they already had a strong grounding in the fundamentals of the biological concepts used in the class, and that the instructors thoroughly explained new terminology. Students stated that “Everything we didn’t know was explained very well” and that, more than learning new terminology, they valued learning “about new applications of the knowledge most of us had already been taught which introduced new viewpoints.” Considering 83 % of student indicated that the final project was the aspect of the course they were most looking forward to, it is not surprising that the metric assessing introduction to new topics and concepts showed a demonstrable increase over the semester between the mid- and end-course surveys. Some students indicated that learning new skills that are not a part of standard bioengineering curriculum, such as CAD design and 3D printing, was an important motivator driving enthusiasm regarding the final project. Students also cited the ability to “use some creativity with our designs” as a source of excitement, indicating that the open-ended and ill-structured nature of the final design problem was a source of motivation for the students.

**Fig. 4:**
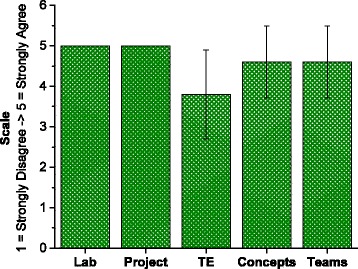
End-Course Survey Results. Student responses to first five questions listed in Table 4 specifically pertaining to class format

The metric that showed the most significant increase between the mid- and end-course time points concerned assessing whether the format of the course was appropriately suited to the material being taught. Responses to specific questions regarding the format of the class, listed in Table 4, are presented in Fig. 4 and Additional file 4: Table S4. The results indicate that the students were already quite familiar with the main theoretical concepts underlying the field of tissue engineering. Since tissue engineering is a well-developed sub-field of the broader discipline of biomedical engineering, it is unsurprising that a group of senior undergraduates pursuing bioengineering majors would report familiarity with the field. However, student responses also show that the labs helped strengthen understanding of tissue engineering concepts by teaching practical lab techniques applicable to this field. Students stated that core concepts are “much easier to understand when the class is structure more hands on, where we can discuss the concepts and then actually implement them ourselves.” Furthermore, 100 % of the students indicated that the labs leading up to the final project helped them understand the goals of the capstone and main course objectives better, stating “I’m enjoying how each experiment we do on a weekly basis is building up to ultimately building a bio-bot”. The first four labs thus served as practice in versatile experimental techniques, and the cumulative nature of the final project proved to be a fair assessment of the training they received.

During the mid-course survey, the students reported lowest satisfaction with the process of writing lab reports as a method of developing a deeper understanding of course material. Students expressed specifically that they would “like more help with the data analysis”. An important objective of this class, in addition to teaching the design principles of biofabrication, was to establish a solid foundation in practicing the experimental method, as students specifically expressed a desire to “have a much stronger ability to design experiments for cell-culture applications in the future” and expected to “learn both lab techniques as well as the best way to plan out experiments and all the variables necessary to take into account”. To address these concerns, the course format was altered to include 20 min of lab discussion time the week before the due date for each lab report. Student teams presented their results to all the instructors, as well as the other teams, and were given the opportunity to discuss their results prior to writing the lab report. Portions of the lab sessions were also allotted to instructor-led data analysis on practice data sets, followed by guided team-based analysis of data generated during the labs. This led to an increase in the level of satisfaction students reported with the lab reports by the end of course survey, with students specifically stating that the in-class presentations “helped clear up any misunderstandings and forced me to think about why we were doing what we did in lab” and were valuable because of their “informal, discussion-based format”. By the end of the semester, 50 % of the students indicated that the lab reports and in-class presentations were the most useful aspects of the course (the other 50 % cited lab work), and 40 % of students cited lab reports as the aspect of the course that helped them learn most efficiently (the other 60 % cited lab work).

The capstone project was intentionally designed to be open-ended, requiring that students form a hypothesis and devise a robust experiment to test that hypothesis using the techniques taught in previous labs. Specifically, students were asked to design and build a biological machine to achieve a functional behavior relevant to an application in biomedical engineering. The student teams, which were self-assigned, reported no conflicts over the course of the semester. Indeed, two of the teams coalesced to form a larger team for the capstone project, in order to test more sample sets and generate more data. Some students attempted to make the walking bio-bots developed in Lab 4 walk at greater speeds by incorporating flexible 3D printed hinges into the bio-bot skeletons. Other students designed an entirely new 3D printed skeleton, mimicking the natural architecture of the esophagus in the human body, to create a muscle-powered peristaltic pump that could potentially be used as an implant for applications in regenerative medicine. Another group of students incorporated skills taught in other bioengineering undergraduate classes, such as genetic engineering of cells and bioinstrumentation, to incorporate new functionalities into the biological machines they had learned to fabricate. The student projects thus displayed broad diversity in terms of target application, while still maintaining a core focus in biofabrication. This served as additional validation that the capstone design project provided students with the unique opportunity to freely pursue empirical inquiry in the field of biomedical engineering and understand the underlying design rules and principles of “building with biology”.

Students were actively engaged in the final project throughout the semester, and presented their ideas for the capstone to instructors prior to the scheduled start of the final lab. The layered roles of the primary instructor with expertise in teaching fundamental biological concepts, the guest lecturer specializing in the topic area of biofabrication and biological machines, and the teaching assistant well versed in the experimental techniques, provided students with different types and levels of individual mentorship and attention, which has been shown to generate positive learning outcomes [3].

By the end of the semester, 100 % of the students in the class indicated that the course fulfilled their expectations, that they enjoyed the course experience, and that they would recommend the course to other students. Additionally, all the student presented their capstone design projects to professors and graduate students in the department working in relevant research fields, and 50 % of the students in the class signed up to continue working on their final projects the following semester under the guidance of the guest lecturer and course instructor. As ethics training is an especially important component of biomedical engineering education [19, 20], all students were required to participate in a discussion on the ethics of building with biology during one of the regularly scheduled class lectures. During this discussion, students discussed vignettes of specific scenarios involving novel biofabrication technology with other students and the instructors [21]. In future iterations of this class, we would like to formally assess how student perceptions and understanding of the ethics of building biological machines shift over the course of the class, in order to improve the quality and focus of our undergraduate bioengineering ethics education programming.

## Conclusions

The rapidly evolving field of biomedical engineering requires adaptive teaching approaches that teach students how to target and solve multi-pronged and ill-structured problems at the cutting edge of scientific research. This is especially important in interdisciplinary fields such as biomedical engineering, as they are subject to many types of conflicting constraints, and require problem-solvers that are well versed in a variety of relevant topics while maintaining depth of expertise in specific focus disciplines. One such discipline that is widely applicable across the field of biomedical engineering, as well as the adjacent fields of mechanical, materials science, and chemical engineering, is that of biofabrication – using modern manufacturing technologies (such as 3D printing) to design and build with biological materials. This course provided students with a foundation in the core design rules and principles of “building with biology”, coupled with specific tools and techniques that were broadly applicable to engineering living tissue for a variety of applications.

The lab-based format of this course, building towards the cumulative yet open-ended final capstone design project, proved to be an effective method of translating the latest biomedical research from the lab bench to the classroom. Incorporating in-class discussion of lab results as preparation for writing lab reports, motivated by mid-course student survey feedback, was shown to enhance student learning outcomes. This format of four relatively well-structured labs leading to a final open-ended project is modular and can readily be adapted to other interdisciplinary courses. In future iterations of the course, we plan to extend the length of time allotted for the final independent project, allowing student teams to truly experience the iterative and adaptive nature of scientific study design. Furthermore, we plan to evaluate the next cohort of students in this course alongside students in more traditional lab classes in the field of biological research. We believe this future study will provide more evidence that the skills-building and independent thinking encouraged by the format of this course directly resulted in improved learning outcomes.

We have shown that a lab-focused structure and layered roles of individual student mentorship can be an efficient and effective method of teaching students to employ the scientific method for designing and executing robust experiments. We anticipate that nurturing these skillsets in undergraduate education will generate engineers who are well prepared for future careers in interdisciplinary fields.

## Additional files

Additional file 1: Table S1. Detailed Course Schedule. (DOC 145 kb) Additional file 2: Table S2. Lab Supply Inventory. (DOC 45 kb) Additional file 3: Table S3. Responses to Rating Scale Questions in Mid- and End-Course Surveys. (DOC 29 kb) Additional file 4: Table S4. Responses to Rating Scale Questions in End-Course Survey. (DOC 30 kb) Additional file 5: Table S5. Responses to Open-Ended Questions in Mid-Course Survey. (DOC 31 kb) Additional file 6: Table S6. Responses to Open-Ended Questions in End-Course Survey. (DOC 30 kb)
