# Supplementary material for: Design and integration of a problem-based biofabrication course into an undergraduate biomedical engineering curriculum
Source: J Biol Eng. 2016 Sep 21;10:10. doi: 10.1186/s13036-016-0032-5 (PMC5031296; doi:10.1186/s13036-016-0032-5)
Supplement: Additional file 1: Table S1. — Detailed Course Schedule. (DOC 145 kb) [file 13036_2016_32_MOESM1_ESM.doc]

**Additional file 1: Table S1: Detailed Course Schedule**
