# Supplementary material for: Design and integration of a problem-based biofabrication course into an undergraduate biomedical engineering curriculum
Source: J Biol Eng. 2016 Sep 21;10:10. doi: 10.1186/s13036-016-0032-5 (PMC5031296; doi:10.1186/s13036-016-0032-5)
Supplement: Additional file 2: Table S2. — Lab Supply Inventory. (DOC 45 kb) [file 13036_2016_32_MOESM2_ESM.doc]

**Additional file 2: Table S2: Lab Supply Inventory**

|  | **Item** | **Amount** |
| --- | --- | --- |
| **Lab Supplies** | 5- 25 mL serological pipettes | 4 cases |
|  | 6, 96 well plates | 1 case |
|  | 15 & 50 mL conical tubes | 2 cases |
|  | 10, 200, 1000 uL pipette tips | 4 cases |
|  | Forceps | 2 sets |
| **Reagents** | Dulbecco's Modified Eagle Medium | 10 500 mL bottles |
|  | Fetal Bovine Serum | 1 500 mL bottle |
|  | Horse Serum | 1 500 mL bottle |
|  | Penicillin/Streptomycin | 1 100 mL bottle |
|  | L-Glutamine | 1 100 mL bottle |
|  | Trypsin-EDTA | 4 50 mL bottles |
|  | PBS w/o Ca, Mg | 10 500 mL bottles |
|  | Long(R) R3 IGF | 0.1 mg |
|  | 6-Aminocaproic Acid | 25 g |
|  | Collagen | 30 mg |
|  | Fibrinogen | 1 g |
|  | Thrombin | 1 KU |
|  | Matrigel | 2 50 mL bottles |
|  | Ethanol | 1 1L bottle |
|  | 2-propanol | 1 4L bottle |
|  | Poly (ethylene glycol) diacrylate | 1 100 mL bottle |
|  | Poly-L LysineHBr | 5 mg |
|  | Lipofectamine 3000 | 0.1 mL |
|  | LDH Cytotoxicity kit | 2 |
|  | CellTiter 96 Viability Assay | 2 |
|  | Live/Dead Viability Stain Kit | 1 |
|  | DAPI | 1 mL |
|  | Texas Red Phalloidin | 1 |
|  | NIH C2C12 murine myoblasts | 1E6 cells |
| **Total Cost of approximately $5000 for class size of 8 students, not including fixed costs such as biosafety hoods, pipettors, safety glasses, etc. or facility maintenance costs.** | | |
|  | | |
