# Supplementary material for: Design and integration of a problem-based biofabrication course into an undergraduate biomedical engineering curriculum
Source: J Biol Eng. 2016 Sep 21;10:10. doi: 10.1186/s13036-016-0032-5 (PMC5031296; doi:10.1186/s13036-016-0032-5)
Supplement: Additional file 3: Table S3. — Responses to Rating Scale Questions in Mid- and End-Course Surveys. (DOC 29 kb) [file 13036_2016_32_MOESM3_ESM.doc]

**Additional file 3: Table S3: Responses to Rating Scale Questions in Mid- and End-Course Surveys**

| Metrics | 1 2 3 4 5  Strongly Disagree -> Neutral -> Strongly Agree |
| --- | --- |
| I expect/expected the course to introduce me to new topics and concepts | MID: 4,4,4,4,5,5,5 |
|  | END: 4,4,5,5,5 |
| The format of the course seems to be/was appropriate | MID: 4,5,5,5,5,5,5 |
|  | END: 5,5,5,5,5 |
| The lab reports are/were helpful for understanding the course material | MID: 3,4,4,4,5,5,5 |
|  | END: 4,4,4,5,5 |
