# Supplementary material for: Design and integration of a problem-based biofabrication course into an undergraduate biomedical engineering curriculum
Source: J Biol Eng. 2016 Sep 21;10:10. doi: 10.1186/s13036-016-0032-5 (PMC5031296; doi:10.1186/s13036-016-0032-5)
Supplement: Additional file 4: Table S4. — Responses to Rating Scale Questions in End-Course Survey. (DOC 30 kb) [file 13036_2016_32_MOESM4_ESM.doc]

**Additional file 4: Table S4: Responses to Rating Scale Questions in End-Course Survey**

| Metrics | 1 2 3 4 5  Strongly Disagree -> Neutral -> Strongly Agree |
| --- | --- |
| Labs leading up to the final project helped me understand the final project better | 5,5,5,5,5 |
| I felt well prepared for the final project presentation | 5,5,5,5,5 |
| I feel that this course contributed greatly to my training in tissue engineering | 2,4,4,4,5 |
| I believe the lab based format helped me learn tissue engineering (TE) concepts better than lecture based format | 3,5,5,5,5 |
| I found value in working in teams on the labs and final project | 3,5,5,5,5 |
| I would recommend this course to other students | 4,5,5,5,5 |
| I enjoyed my experience in this course | 4,5,5,5,5 |
| The course fulfilled my expectations | 4,4,5,5,5 |
