# Supplementary material for: Design and integration of a problem-based biofabrication course into an undergraduate biomedical engineering curriculum
Source: J Biol Eng. 2016 Sep 21;10:10. doi: 10.1186/s13036-016-0032-5 (PMC5031296; doi:10.1186/s13036-016-0032-5)
Supplement: Additional file 5: Table S5. — Responses to Open-Ended Questions in Mid-Course Survey. (DOC 31 kb) [file 13036_2016_32_MOESM5_ESM.doc]

**Additional file 5: Table S5: Responses to Open-Ended Questions in Mid-Course Survey**

| Metrics | Please explain/expand here. |
| --- | --- |
| What aspects of the course are you most looking forward to? | - I am excited for the final project. - I’m enjoying how each experiment we do on a weekly basis is building up to ultimately building a biobot. - Learning how to do CAD to design out biobot skeletons. - I’m excited about the final project because we’ll get to use some creativity with our designs. - Making a bio-bot! This will be exciting. - I’m most looking forward to when we place the muscle cells and create the biobot. |
| Do you want the biology to be explained more explicitly in the context of the material being taught? | - No, I feel as though I already understand the biology referenced. - No, Pablo explains everything we don’t know, and understands that we know other things enough. - No, I think the amount that each subject covered is good – we have heard a lot of this material already but it’s good to get a refresher as well as discuss how it applies to what we’re doing. - No, much of the biology we have already had (or had multiple times). It is good to do a review in lectures, but the level of detail has been fine. - I think the biology presented to us is adequate for understanding what we’re doing in the lab; we have a really strong biology background to start with so not a ton of explanation is always necessary. - No, we have learned all the biology before. Just a brief review is enough. - I understand the basic biology. The only thing is sometimes I am not sure what each reagent does on the molecular level. |
| What are your thoughts about the level at which this course is being taught? (too advanced, just right, other) | - Just right - Just right - Just right – we are getting an overview in class and then meeting twice in lab which is not too much. - Not advanced/just right. Lab reports should be graded fairly stringently, we are all graduation and should be able to write well at this point. - This is a good age level for this lab, although I think it might be a better class for juniors because you would be able to transition more easily from our BIOE 202 cell culture class that we take as a sophomore. I haven’t had any issues transitioning because I do alot [sic] of this stuff in my research lab on campus, but I think others struggled at the beginning of the class due to that gap between sophomore and senior year. - I don’t know in lab techniques are extremely simple, but I would like more help with the data analysis parts… - I think it is just right. It is a good balance of lecture and lab work. |
