# Supplementary material for: Design and integration of a problem-based biofabrication course into an undergraduate biomedical engineering curriculum
Source: J Biol Eng. 2016 Sep 21;10:10. doi: 10.1186/s13036-016-0032-5 (PMC5031296; doi:10.1186/s13036-016-0032-5)
Supplement: Additional file 6: Table S6. — Responses to Open-Ended Questions in End-Course Survey. (DOC 30 kb) [file 13036_2016_32_MOESM6_ESM.doc]

**Additional file 6: Table S6: Responses to Open-Ended Questions in End-Course Survey**

| Metrics | Please explain/expand here. |
| --- | --- |
| Which aspect of the course helped you learn most efficiently? | - Lab Reports - Lab Work - Lab Work - Lab Work - Presentations |
| What aspects of the course did you find most useful? | - The lab reports and weekly presentations were very useful. They helped clear up any misunderstandings and forced me to think about why we were doing what we did in lab. - Working in the lab was most helpful. The concepts were explained in class but it’s much easier to understand when the class is structure more hands on where we can discuss the concepts and then actually implement them ourselves. - Writing the lab reports was probably the most useful. There’s where you really have to know your stuff to write it well. - Reviewing results in class in a more informal, discussion-based format. |
| Was the biology explained explicitly enough to assist you with the other material being taught? | - Yes - Yes. Everything we didn’t already know was explained very well. - Yes, the biology refreshers were explained well although none of the biology discussed was new information to me. It was more about new applications with the knowledge that most of us had already been taught which introduced new viewpoints. - Yes. We have had all the biology before. - Yes, built upon concepts presented in other courses. |
| Provide your thoughts about the level at which this course was taught (too advanced, just right, other) | - Just right - Just right - Just right – it wasn’t too difficult and if we were having trouble with anything then we could always reach out for help and get extensions if other things were going on. The course was flexible so it was able to be at our pace. - I think that the level of work and reports etc was good. However, if you are extremely experience [sic] in cell culture already, it didn’t teach you all that much by being in lab. - Just right – high, but reasonable expectations. |
